# Effects of Drug Administration on Postoperative Pain in Orthognathic Surgery: A Systematic Review

**DOI:** 10.1002/cre2.70226

**Published:** 2025-09-21

**Authors:** Gianna Dipalma, Angelo Michele Inchingolo, Lilla Riccaldo, Roberta Morolla, Pietro Lauria, Roberto Vito Giorgio, Valeria Colonna, Benito F. P. Pennacchio, Antonio Mancini, Andrea Palermo, Alessio Danilo Inchingolo, Francesco Inchingolo

**Affiliations:** ^1^ Department of Interdisciplinary Medicine University of Bari “Aldo Moro” Bari Italy; ^2^ Department of Biomedical, Surgical and Dental Sciences Milano University Milan Italy; ^3^ Università del Salento, University of Salento‐Dipartimento di medicina sperimentale Italy

**Keywords:** maxillofacial surgery, orthognatic surgery, postoperative pain

## Abstract

**Objectives:**

This systematic review aims to offer indications on how drug administration affects postoperative pain in patients undergoing orthognathic surgery.

**Material and Methods:**

We searched Pubmed, Scopus, and Web of Science for articles pertaining to our research topic. The search strategy was created by combining terms associated with our review's goal, which is to ascertain whether drugs used before, during, and after surgery help lessen postoperative pain. The keywords used were "orthodontic surgery AND postoperative pain." Inclusion criteria used to select articles were human subjects only, open‐access studies, clinical and randomized controlled trials, English language, and studies published no more than 10 years before the current edition. Exclusion conditions such as eta‐analyses, systematic reviews, off‐topics, abstracts, posters, and public trials were excluded.

**Results:**

Thirteen randomized control trials and one comparative study comprised the design of the chosen research. The studies show that administering a certain drug in comparison to another can lessen the severity of postoperative sequelae.

**Conclusions:**

A multimodal approach is needed to alleviate post‐discomfort after orthognathic surgery. Medication given before surgery can lessen discomfort and the need for opioids. Pain intensity can be decreased, and early opioid tolerance can be avoided with intraoperative treatments. Non‐steroidal anti‐inflammatory drugs and iontophoresis‐based transdermal medication administration are other alternatives.

**Abbreviations**
BIMAX: bimaxillary
BOS: bimaxillary orthognatic surgery
BSSO: bilateral sagittal split osteotomy
BSSRO: bilateral sagittal split ramus osteotomy
CRP: C‐reactive protein
DEX: dexmedetominide
F‐F grp: fentanyl and fentanyl group
GLM: general linear model
IM: intramuscular
IV: intravenous
NLR: neutrophillymphocyte ratio
NMDA: *N*‐methyl‐d‐Aspartate
NSAIDs: non‐steroidal anti‐inflammatory drugs
OGS: orthognatic surgery
PO: postoperative
PON: postoperative nausea
PONV: postoperative nausea and vomiting
POP: postoperative pain
R‐F grp: remifentanil and fentanyl group
R‐R grp: remifentanil and remifentanil group
VAS: visual analog scale
WBC: white blood cell count

## 1 Introduction

Orthognathic procedures are regarded as the cornerstone of treatment for complex malocclusions, jaw discrepancies, and preserving a healthy and stable dentoskeletal connection (Ansari et al. 2022; Mohammadi et al. 2016).

This surgical treatment often leads to postoperative pain (POP) and edema because it involves muscle stripping, tissue manipulation, and repeated osteotomies (Ansari et al. 2022; Mohammadi et al. 2016; Samieirad et al. 2018; Labafchi et al. 2023; Tatullo et al. 2012).

Most of the time, patients are uncomfortable because of POP and edema (Ansari et al. 2022; Mohammadi et al. 2016; Samieirad et al. 2018; Mobini et al. 2018).

High rates of reported patient dissatisfaction are linked to poorly controlled POP (Ansari et al. 2022; Samieirad et al. 2018; Labafchi et al. 2023; Dadmehr 2022).

Managing POP following orthognathic surgery (OGS) effectively is a difficult task (Samieirad et al. 2018; Phillips et al. 2015; Inchingolo et al. 2014; Yang et al. 2024; Raokadam et al. 2024; Huang et al. 2024; Chan 2024; Abdelemam 2024; Yurdakurban et al. 2024; Wang and Zhang 2024; Uzunçıbuk 2024; Minervini et al. 2023a, 2023b, 2023c, 2023d, 2023e, 2023f).

Serious surgical complications such wound dehiscence or surgical site bleeding (Rossi et al. 2023; Cantore et al. 2014; Inchingolo et al. 2020), hematoma, aspiration and choking risk, and hydration and electrolyte abnormalities can ensue from postoperative nausea and vomiting. These issues can also cause delayed hospital discharge and higher healthcare expenses (Phillips et al. 2015; Brookes 2015; Silva et al. 2006; Maitra et al. 2016; Shilpa et al. 2015; Bano 2008; Apfel et al. 1999; Balzanelli et al. 2022; Oelerich et al. 2024; Marino Merlo et al. 2024; Isatsu et al. 2024; Gandedkar et al. 2024; Aftabi et al. 2024; Aulia 2022; Patel 2024; Jara 2024; Cordeiro et al. 2024; Molins et al. 2024; Inchingolo et al. 2010, 2011a, 2012; Dang 2021).

Improving pain relief and maintaining hemodynamic stability before and after OGS are therefore critical steps in the early postoperative (PO) phase (Ansari et al. 2022; Mohammadi et al. 2016; Samieirad et al. 2018; Dadmehr 2022; Eshghpour et al. 2018; Inchingolo et al. 2022c; Balzanelli et al. 2021; Y. Li et al. 2024; Xu et al. 2024; Karami et al. 2024; Sharma et al. 2024; Alpaydin et al. 2024; Guo et al. 2024; Laconi et al. 2024; Azher et al. 2024; Bambini et al. 2017, 2005; Giorgini 2017; Bambini et al. 2005).

Preoperative pain, anxiety, age, and the type of surgery are all said to have an impact on POP, and the factors that influence the amount of analgesics used after surgery include age, psychological distress, and the type of operation (Ip et al. 2009; Aoki et al. 2014; Ballini 2020; Inchingolo et al. 2022b; Malcangi et al. 2022). Patients will benefit from more efficient pain treatment if preoperative and intraoperative factors are clarified and controlled (Aoki et al. 2014; Inchingolo et al. 2021a; D'Esposito 2020; Caggiano 2022; Gasparro 2019; Sammartino et al. 2005; Mortellaro 2008; Piombino et al. 2015)/span > .

Preemptive analgesia is based on the idea that improved POP management initiates the analgesic procedure before the surgical incision, during the preoperative phase (Canpolat et al. 2021; Ballini 2019). In particular, the pre‐emptive analgesia concept—which incorporates both peripheral and cerebral sensitization—makes up the pathophysiology of surgical pain (Inchingolo et al. 2021a; Canpolat et al. 2021). By causing hyperexcitability in the spinal cord posterior horn neurons via afferent C fibers, the nociceptive sensations brought on by the afferent impulses of the wound site create central sensitization (Inchingolo et al. 2021b; Signorini 2021).

Patients are saved from POP by performing preemptive analgesia (Canpolat et al. 2021; Ballini 2015). Preventive analgesia, on the other hand, is a more recent term for POP management that encompasses any treatment plan that is administered before, during, or after surgery to manage pain‐induced sensitization (Canpolat et al. 2021; Wall 1988; Phillips 2008; McQuay 1995; Crile 1913; Hurley and Wu 2010). The comprehensive concept of preventive analgesia has contributed to its recent rise in popularity (Canpolat et al. 2021; Inchingolo et al. 2022a).

Preemptive and preventive analgesia both lessen acute pain, which in turn keeps it from turning into chronic pain (Canpolat et al. 2021; Møiniche et al. 2002; Hemati et al. 2023).

It appears that the use of pharmaceutical analgesic therapies is crucial for minimizing POP (Ansari et al. 2022; Agbaje et al. 2018; Inchingolo et al. 2023).

Opioids are frequently recommended to treat moderate‐to‐severe pain after surgical operations because of their central effects (Ansari et al. 2022; Malcangi et al. 2023; Arrigoni 2022). The most commonly used agents in this setting include morphine, fentanyl, oxycodone, and tramadol. These drugs are often administered intravenously or through patient‐controlled analgesia (PCA) pumps in the immediate postoperative phase. While they provide strong pain relief, they are associated with relevant adverse effects such as sedation, respiratory depression, nausea, vomiting, and the potential for tolerance or dependence. Therefore, their use in orthognathic surgery is now frequently limited to short‐term protocols or combined with non‐opioid drugs in multimodal analgesic strategies (Ansari et al. 2022; Samieirad et al. 2018; Agbaje et al. 2018; Inchingolo et al. 2022d; Rapone 2022; Grassi et al. 2011; Rapone et al. 2023).

Adjuvant nonopioid analgesics have also shown promise in lowering pain following surgery (Ansari et al. 2022; Samieirad et al. 2018; Agbaje et al. 2018; Fay et al. 2024; Baydan and Soylu 2024; Payman et al. 2024; N. Li 2024; Ni et al. 2024; Hua et al. 2024; Kämmerer et al. 2024; Adamska et al. 2024; Musa et al. 2024; Dana et al. 2024; Kılınç and Mansız 2024; Chhabrani et al. 2024; Topbaş 2024; Zhang and Li 2024; X. Liu et al. 2024; X. Lei et al. 2024). Recent advances in pharmacological management of POP after orthognathic surgery increasingly emphasize the use of non‐opioid agents, either alone or in combination with opioids, to optimize pain control while limiting adverse outcomes (Payman et al. 2024; N. Li 2024; Ni et al. 2024; Hua et al. 2024; Kämmerer et al. 2024; Adamska et al. 2024; Musa et al. 2024; Dana et al. 2024; Kılınç and Mansız 2024; Chhabrani et al. 2024; Topbaş 2024; Zhang and Li 2024; X. Liu et al. 2024; X. Lei et al. 2024). Nonsteroidal anti‐inflammatory drugs (NSAIDs) such as ibuprofen and ketorolac, as well as acetaminophen, are widely adopted to decrease inflammation and pain while reducing the need for opioids. More recently, selective COX‐2 inhibitors like celecoxib have been explored due to their lower gastrointestinal side‐effect profile. Additionally, anticonvulsants such as pregabalin and gabapentin have been tested as preoperative agents to reduce central sensitization, showing efficacy in lowering acute pain scores and opioid consumption (Canpolat et al. 2021; Møiniche et al. 2002; Hemati et al. 2023). Protocols frequently involve combining opioids with NSAIDs or acetaminophen in a multimodal fashion, administered preoperatively or intraoperatively, to maximize analgesic benefit while minimizing risks. These strategies represent a current trend in post‐orthognathic surgery pain management (Woolf 2011; Ferrazzano et al. 2006; Alimoradi et al. 2024; Q. Lei et al. 2024; Owens 2024; Long et al. 2024; Dammling et al. 2024; Kim 2024; Altwaijri 2024; Habib et al. 2024; Y. Liu 2024; Isacco 2021; Goldoni 2022). The administration of opioid drugs must be done so with caution and judgment due to the difficulties connected with opioid consumption, such as euphoria, tolerance, addiction, and drug overdose (Ansari et al. 2022; Samieirad et al. 2018; Agbaje et al. 2018; Inchingolo et al. 2022d; Rapone 2022; Grassi et al. 2011).

It is important to remember that one of the risk factors for postoperative nausea and vomiting (PONV) is opioid use. Overall, the shift in recent years has been toward preventive and multimodal analgesia protocols, in which different drug classes are combined according to specific timing (preoperative, intraoperative, or immediate postoperative). This approach has demonstrated improvements in patient comfort, faster recovery, and reduced hospital stay. However, despite encouraging results, there is still no consensus on which pharmacological protocol is most effective, and further high‐quality evidence is required (Ansari et al. 2022; Rapone et al. 2023).

Which method and which agent would be better suitable for applying pre‐emptive analgesia are currently being debated. It is still unknown which agent or approach is better than the others (Canpolat et al. 2021; Woolf 2011; Ferrazzano et al. 2006; Alimoradi et al. 2024; Q. Lei et al. 2024; Owens 2024; Long et al. 2024; Dammling et al. 2024; Kim 2024; Altwaijri 2024; Habib et al. 2024; Y. Liu et al. 2024; Isacco 2021; Goldoni 2022).

After surgery, effective acute pain management is essential to inpatient care because it facilitates a simple recovery and hospital discharge (Ansari et al. 2022; Samieirad et al. 2018; Mobini et al. 2018; Dadmehr 2022; Panula et al. 2001; Inchingolo et al. 2011b; Bavetta 2019).

**Figure 1:**
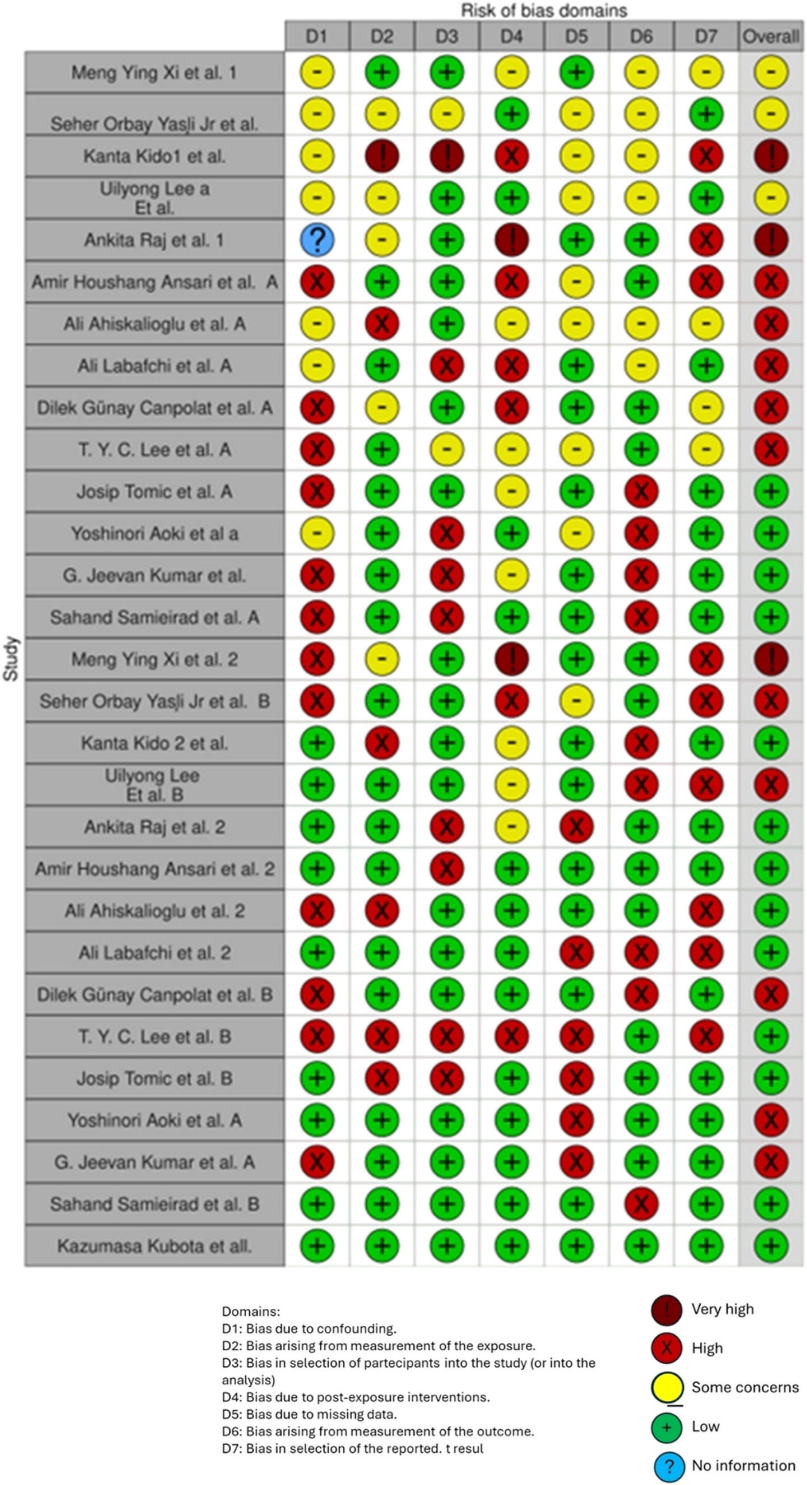
Summary of the aim of the article.

The aim of this systematic review is to evaluate and compare the effectiveness of drug administration in the preoperative, intraoperative, and PO phase of orthognathic surgical procedures (Figure 1).

## 2 Materials and Methods

### 2.1 Protocol and Registration

This study adhered to the Preferred Reporting Items for Systematic Reviews and Meta‐Analyses (PRISMA) guidelines and was registered with PROSPERO (International Prospective Register of Reviews) under registration number 541452 (Page 2021).

### 2.2 Search Development

Pubmed, Scopus, and Web of Science searches were conducted to find articles related to our research topic covering the period from January 1, 2013, to March 19, 2024. The search methodology was developed by combining terms relevant to the objective of our review, which is to determine whether pre, during, and postoperative medications can reduce POP. Accordingly, the following binary variables and keywords were used, “orthodontic surgery AND POP.”

### 2.3 Inclusion Criteria

The following inclusion criteria were used to select articles: human subjects only, open‐access studies, clinical and randomized controlled trials, English language, and studies published no more than 10 years before the current edition. Randomized controlled trials that compared the use of certain drugs with a placebo before, during, and after surgery; as well as controlled studies that used iontophoresis as a method of subtotal drug delivery after surgery.

### 2.4 Exclusion Criteria

Meta‐analyses, systematic reviews, off‐topics, abstracts, posters, and public trials were excluded.

Other exclusion criteria were research published more than 10 years before the current study, research conducted in a foreign language, research with limited access, and research conducted on animals or in vitro.

### 2.5 Data Processing

Four reviewers conducted database searches to illustrate studies and independently assess their quality according to previously established inclusion and exclusion criteria. Articles that did not meet the requirements for manuscript and abstract were excluded during the screening phase. The full texts of the remaining articles were read to conduct an eligibility analysis. The selected articles were published in Mendeley (version 2.112.2).

### 2.6 Data Processing

The PICO question is “What medications contribute to the decrease of POP in OGS?” (Table 1).

**Table 1:** The components of the PICOS criteria (population, intervention, comparison, outcome, study design), which include population, intervention, comparison, outcome, and research design, as well as their use in this evaluation.

Population	Subjects undergoing oral and maxillofacial surgery
Intervention	Orthognatic surgery (OGS)
Comparison	Reduction of POP after medication before, during, and after oral and maxillofacial surgery
Outcome	Best way to reduce postoperative pain
Study design	Clinical trials, randomized controlled trials

### 2.7 Quality Assessment

The quality of the included papers was assessed by two reviewers, A.M.I. and A.D.I., using the ROB‐2 is a tool developed to assess risk of bias in the results of non‐randomized studies that compare health effects of two or more interventions. Seven points were evaluated, and each was assigned a degree of bias. A third reviewer (F.I.) was consulted in the event of a disagreement until an agreement was reached. The question in the domains evaluated in the ROB‐2 is the following:

– Bias due to confounding.
– Bias arising from measurement of exposure.
– Bias in the selection of participants into the study.
– Bias due to postexposure intervention.
– Bias due to missing data.
– Bias arising from measurement of the outcome.
– Bias in the selection of the reported results.

## 3 Results

**Figure 2:**
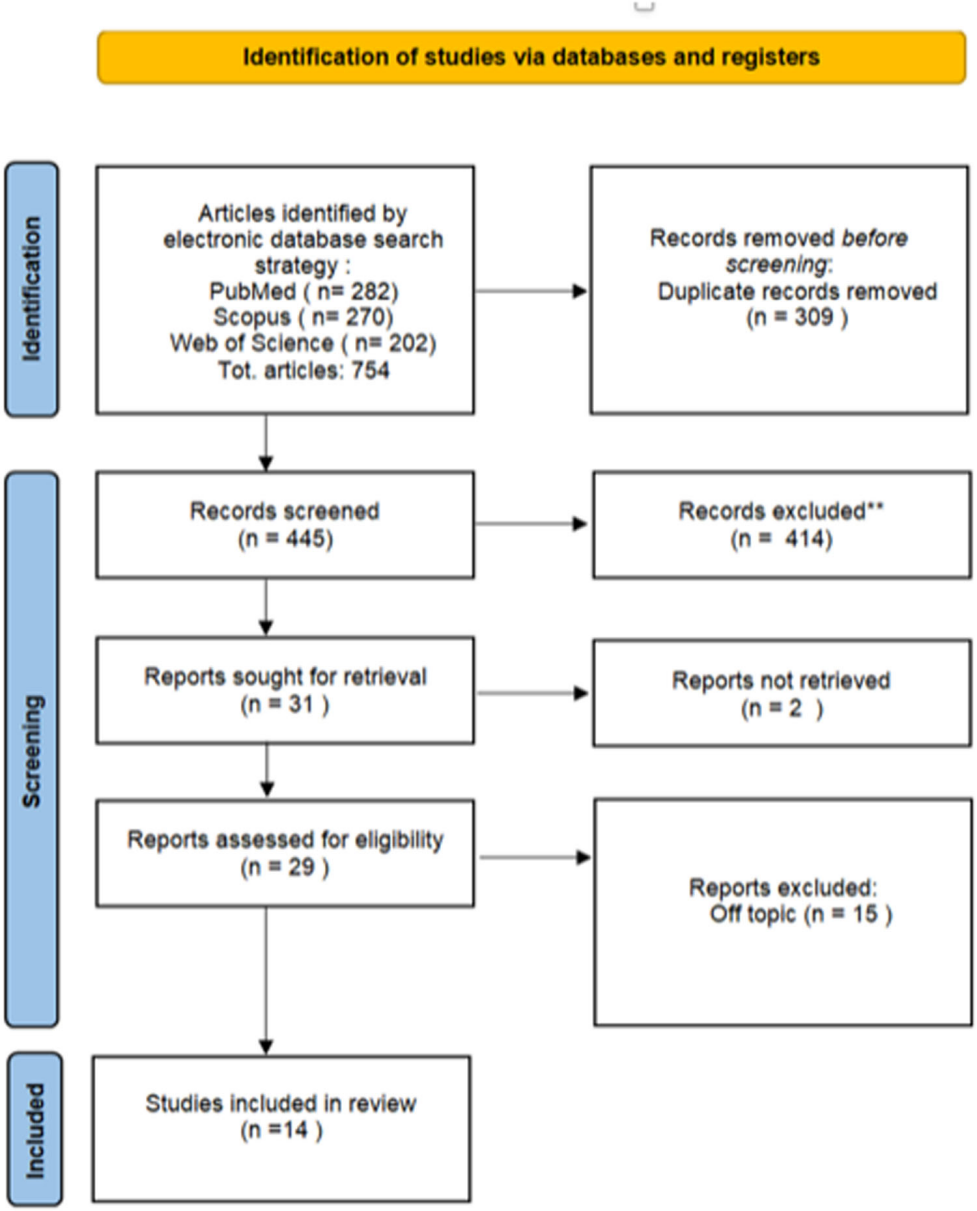
PRISMA ScR flowchart diagram of the inclusion process.

The selection process is summarized in Figure 2. The electronic database search identified a total of 754 articles (Scopus *N* = 270, PubMed *N* = 282, Web of Science *N* = 202), and no articles were included through manual search. In a first step, 309 articles found to be duplicates were removed, and then 445 articles were screened for title and abstract. In total, 414 articles were not selected after abstract screening, mainly because they dealt with a type of maxillofacial surgery that was not of interest to us and because they were systematic reviews. Thus, 31 articles were selected for eligibility evaluation. Subsequently, 2 articles were excluded because they were not available on any platform and not free. Of the remaining 29, 15 were eliminated after full‐text evaluation because they were off topic. Finally, 14 articles were selected for systematic review. The design of the selected studies included 13 Randomized Control Trials and 1 comparative study. All selected studies analyzed the method to be able to decrease the extent of POP in OGS following the administration of anti‐inflammatory or anesthetic drugs before performing surgery or during surgery. In all of the studies, it can be seen that the administration of some drugs compared to others can decrease the extent of POP, edema, and nausea; while in others, it can be seen that the administration of some drugs allows the avoidance of other drug products that can cause addiction. Beyond the retrieval process, the selected studies provided relevant insights into pain management strategies in orthognathic surgery. Overall, the administration of specific drugs before or during surgery significantly reduced postoperative pain (POP), edema, and in some cases nausea and vomiting.

- **Opioid‐sparing strategies:** Several trials demonstrated that preemptive administration of pregabalin, ketamine, or lidocaine infusion reduced postoperative opioid consumption and improved pain scores, highlighting their role as effective adjuvants in multimodal analgesia.
- **Non‐opioid analgesics:** Ibuprofen was consistently found to provide superior analgesia compared to diclofenac or diclofenac‐orphenadrine combinations, particularly in BIMAX procedures, while single‐dose IV ibuprofen also reduced opioid intake and pain scores. Tenoxicam, alone or combined with paracetamol, showed favorable effects on POP control.
- **Adjuvant therapies:** Melatonin improved not only pain but also neurosensory recovery, whereas montelukast demonstrated anti‐inflammatory properties with significant pain reduction after BOS. Dexmedetomidine reduced both pain and postoperative nausea and vomiting, suggesting benefits beyond analgesia.
- **Local and regional approaches:** Regional anesthesia with ropivacaine nerve blocks was found to be more effective than intramuscular diclofenac for acute pain control. Case reports also showed that lidocaine iontophoresis successfully alleviated pain in patients with respiratory comorbidities.
- **Multimodal regimens:** Combinations such as ondansetron plus dexamethasone or clonidine plus dexamethasone were effective in reducing both POP and postoperative nausea and vomiting, confirming the importance of integrated pharmacological approaches.

Table 2 presents the summary of the records chosen.

**Table 2:** Descriptive summary of item selection.

Authors	Study design	Aim	Number of patient/teeth	Materials and methods	Molecule(s) used	Outcomes
Xi et al. (2020)	Randomized, double‐blind, controlled clinical trial	To determine if nalbuphine offers better postoperative analgesia in OGS than sufentanil, and if this better analgesia is brought about by controlling oxidative stress and inflammation.	60 patients	30 patients in the nalbuphine group (observation group) 30 patients in the sufentanil group (control group). 16 to 28‐year‐olds who were planned to have OGS, which included genioplasty, bilateral sagittal split ramus osteotomy (BSSRO), and Le Fort I osteotomy, all performed under general anesthesia.	Nalbuphine vs. Sufentanil	After OGS, nalbuphine provides superior sedation and postoperative analgesia. Because it lowers oxidative stress and inflammation, nalbuphine also appears to offer better postoperative analgesia.
Kido et al. (2019)	Prospective randomized controlled study	To investigate the potential protective effects of a modest dose of ketamine against remifentanil‐induced acute opioid tolerance and its potential impact on the neutrophil‐lymphocyte ratio (NLR), a recently identified indication of inflammation.	40 patients underwent the BSSRO for mandibular prognathism.	One of three anesthetic regimens—high‐dose remifentanil, low‐dose remifentanil, or high‐dose remifentanil plus ketamine—was administered to participants at random during surgery. Fentanyl was administered intravenously under patient supervision. In the initial 24 h following surgery, fentanyl use and Visual Analog Scale (VAS) pain scores were documented. Levels of NLR and C‐Reactive Protein (CRP) in the serum were also measured perioperatively.	Remifentanil ± Ketamine	Low‐dose ketamine infusion prevented postoperative acute opioid tolerance caused by high‐dose intraoperative remifentanil. After surgery, ketamine elevated the postoperative NLR in correlation with a lower need for fentanyl to manage pain.
Lee et al. (2017)	Prospective, randomized, double‐ blind, placebo‐controlled study	Analyze the impact of Intravenous (IV) lidocaine on POP relief following bimaxillary (BIMAX) surgery.	52 patients underwent BIMAX surgery	Two groups were randomly assigned to the patients. Patients were divided into two groups at random: group L received a bolus of 1.5 mg/kg and an infusion of 2 mg/kg/h, while group C was given normal saline. White Blood Cell (WBC) count, total, and postoperative edema were recorded.	Lidocaine (IV infusion)	During BIMAX surgery, systemic lidocaine infusion lowers analgesic intake, postoperative discomfort, and facial edema.
Raj et al. (2021)	Comparative study	Compare intramuscular (IM) diclofenac versus regional anesthesia for the treatment of POP after maxillofacial surgery.	30 patients underwent bi‐jaw OGS	Group A consisted of 15 patients who had a bilateral inferior alveolar nerve block at the surgical site using 0.5% ropivacaine, while Group B consisted of 15 patients who received 75 mg IM diclofenac right before extubation. Periodically, the pain was assessed at 2, 4, 6, and 12 h after surgery.	Diclofenac vs. Ropivacaine (nerve block)	It has been noted that intraoral nerve blocks, a type of regional anesthesia, are more effective at managing acute POP than IM diclofenac (75 mg).
Ansari et al. (2022)	Triple‐blind, Randomized, clinical trial	To determine how oral montelukast preoperative administration affected the degree of pain after Bimaxillary Orthognathic Surgery (BOS).	60 patients underwent OGS in class III.	Montelukast group (case group) and placebo group (control group), respectively involve fifteen patients. There was no discernible correlation between the patient's age, gender, or BMI and the degree of pain.	Montelukast	To alleviate POP after BOS, preoperative montelukast medication is useful.
Ahiskalioglu et al. (2016)	Prospective, randomized, and double‐blinded study	Examine the impact on PO analgesia and opioid intake in patients undergoing double jaw surgery using a single 150 mg pregabalin preemptive dosage.	40 patients were scheduled for elective double jaw surgery under general anesthesia.	Two groups of patients were randomly assigned to each group: the pregabalin group (20 patients) received 150 mg of pregabalin orally 1 h before general anesthesia, and the placebo group (20 patients) received an oral placebo capsule.	Pregabalin (150 mg)	Within the first 24 h following double jaw surgery, PO opioid use was reduced by a single 150 mg dosage of preemptive pregabalin.
Labafchi et al. (2023)	Triple‐blind, randomized, clinical trial	To evaluate the effectiveness of dexmedetomidine (DEX) in patients who underwent OGS in terms of pain management and prevention of nausea and vomiting.	60 patients with a scheduled BOS for a class III.	The placebo group got normal saline, the DEX group received premedication with DEX 1 mg/kg IV over 10 min, followed by a maintenance dosage (0.2 mg/kg/hour). PO discomfort, nausea, and vomiting were the main outcome variables. A VAS was used to measure pain at 1, 3, 6, 12, 18, and 24 h following surgery.	Dexmedetomidine (IV)	After BOS, premedication with DEX may be a useful therapeutic alternative for decreasing PO nausea and POP.
Canpolat et al. (2021)	Single‐center, prospective, double‐blind, and randomized study	To study the role, on POP management in OGS, of a single‐dose IV ibuprofen administration for preventive analgesia.	40 adult patients underwent BIMAX osteotomy.	Two group, consisting each one of 20 patients, treated with Ibuprofen and placebo respectively.	Ibuprofen (IV)	To prevent PO opioid intake and lower VAS scores in patients undergoing OGS, a single‐dose IV ibuprofen administration was used shortly before the procedure.
Lee and Curtin (2020)	Triple‐blind, randomized, controlled clinical trial	Evaluate the efficacy of melatonin on nerve healing and pain control following OGS	30 orthognathic patients	2 groups received either oral melatonin or an identical placebo as a preventative measure for 21 straight days. Numbness, objective neurosensory function, and subjective pain were among the pre‐ and postoperative clinical evaluations.	Melatonin (oral)	Prophylactic melatonin therapy improves POP and narcotic use, improves sensory recovery after surgery, and leads major clinical advantages.
Tomic et al. (2022)	Prospective, randomized, double‐blind, controlled clinical study	To determine ibuprofen's analgesic efficacy in OGS compared to diclofenac with orphenadrine for POP.	109 patients, 48 of whom had BIMAX and 51 of whom had Bilateral Sagittal Split Osteotomy (BSSO)	OGS patients were randomly assigned to one of two groups and given IV medication twice a day: 600 mg of ibuprofen (I‐group) or 75 mg of diclofenac + 30 mg of orphenadrine (d‐group).	Ibuprofen vs. Diclofenac + Orphenadrine	Patients who had BIMAX osteotomy on the third PO day reported decreased pain when taking ibuprofen and having a lower body mass index. For better pain management following OGS, doctors might therefore favor ibuprofen.
Aoki et al. (2014)	Clinical trial study	To examine the path of fentanyl consumption over time using IV patient‐controlled analgesia records from patients undergoing OGS for mandibular prognathism and to assess the impact of sex, anesthetic, and surgical techniques on the course of consumption.	143 healthy individuals, 56 men and 87 women, aged 15 to 53, was planned to have OGS to treat mandibular prognathism.	Three groups were created: patients in the F‐F grp received fentanyl induction and maintenance; patients in the F‐R grp received fentanyl maintenance; and patients in the R‐R grp received remifentanil induction and maintenance.	Fentanyl/Remifentanil (PCA)	The remifentanil and fentanyl groups received their medication in different ways, although the three groups' combined PO 24‐h intake did not differ. Furthermore, the VAS ratings at three and 24 h were primarily less than 50 mm, and they did not differ between the two anesthetic groups (F‐F grp and F‐R grp), suggesting that subjective pain was adequately managed in both groups.
Samieirad et al. (2018)	Randomized controlled triple‐blind trial.	To compare the effectiveness of ondansetron with dexamethasone versus clonidine with dexamethasone for preventing POP, nausea, and vomiting in patients undergoing OGS.	30 patients with skeletal class III, planned to have OGS, were split into two equal‐number groups at random and given either clonidine or ondansetron.	1 h before surgery, patients were given oral ondansetron 8 mg or oral clonidine 150 μg as premedication (both dissolved in 20 cc of water). Additionally, both groups received 8 mg of dexamethasone IVly every 4 h during the procedure and 1 h prior.	Ondansetron + Dexamethasone vs. Clonidine + Dexamethasone	A premedication regimen combining oral ondansetron and IV dexamethasone may be suggested as a successful multimodal strategy to lessen postoperative severe pain and PONV in orthognathic operations while avoiding serious adverse effects.
Yaşli et al. (2023)	Single‐centered, randomized, double‐blind study	To evaluate the effects of tenoxicam and paracetamol applied separately versus in combination on POP and opioid use in individuals having double jaw surgery.	60 patients	20 patients undergoing double jaw surgery were included in the study. Opioid use was documented after the procedure, and pain severity was reported every 30, 60, 100, and 12 min.	Tenoxicam ± Paracetamol	For PO analgesia, tenoxicam and paracetamol‐tenoxicam combinations—especially the latter—were proven to be useful alternatives.
Kumar et al. (2023)	Randomized, clinical trial	To evaluate the impact of oral montelukast preoperative treatment on the level of discomfort experienced after BOS.	40 patients. Each one has a skeletal class III and has been scheduled a BOS appointment.	A 10‐mg pill of montelukast dissolved in apple juice was given to the intervention group an hour before to surgery, while the placebo group received no medication.	Montelukast	It has been noted that intraoral nerve blocks, a type of regional anesthesia, are more effective at managing acute POP than IM diclofenac (75 mg).
Kubota et al. (2018)	Case report	Two patients with aspirin‐exacerbated respiratory diseases had their POP managed transdermally using iontophoresis by alternating current.	2 patients who had respiratory conditions made worse by aspirin had their mandibular wisdom teeth out and their sagittal ramus osteotomy.	The electrode was made of cotton and a copper wire mesh. The 2 electrodes were dipped in 4% lidocaine and placed separately on the facial skin, after which a pulsed alternating voltage (1 V; 50 Hz) was applied for 30 min or 60 min. To measure pain perception, it was used we a standard VAS scale.	Lidocaine (iontophoresis)	Treatment with montelukast before surgery can considerably lessen pain after BOS. More study is needed to make the results more trustworthy.

## 4 Quality Assessment and Risk of Bias of Included Articles

**Figure 3:**
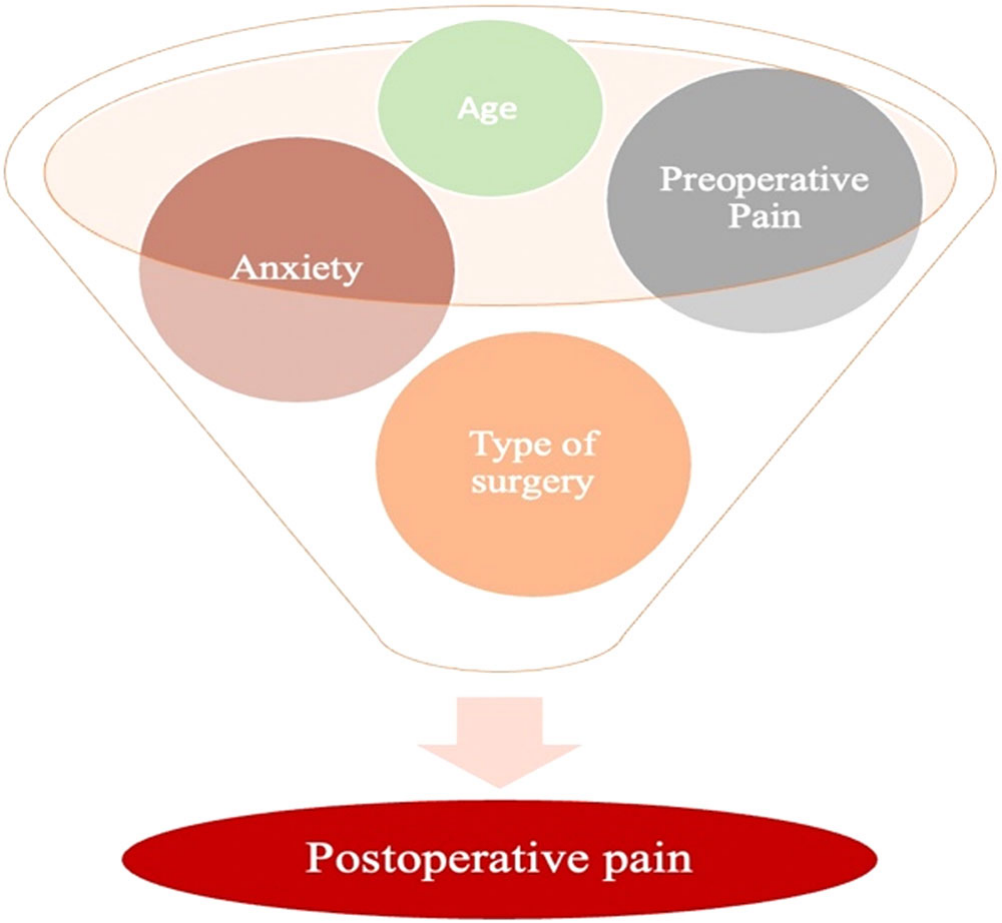
Bias assessment.

The risk of bias in the included studies is reported in Figure 3. Regarding the bias due to confounding most studies have a high risk. The bias arising from measurement is a parameter with low risk of bias. Many studies have low risk of bias due to bias in selection of participants. Bias due to post exposure cannot be calculated due to high heterogeneity. The bias due to missing data is low in many studies. Bias arising from measurement of the outcome is low. Bias in the selection of the reported results is high in most studies. The final results show that 5studies have low risk of bias, 12 have a very high risk of bias and 9 have high risk of bias.

## 5 Discussion

Many patients still experience pain after orthognathic surgery (Shiva Prasad et al. 2008; Goodchild et al. 2017), despite advancements in understanding the pathophysiology of pain, developing new drugs, and improving management strategies. A key element in accelerating surgical recovery and reducing the duration of hospital stay and associated complications is appropriate and sufficient POP (Wang 2004) management achieved with pre, intraoperative, and PO medications.

### 5.1 Medications and Procedures for POP Control Administered Before the Surgery Itself

The article of Samieirad et al. (2018) states that to control nausea, discomfort, and vomiting before oral and maxillofacial surgery (Gan 2014), DEX is often used.

An advantage of DEX as an analgesic is its ability to induce analgesia without producing respiratory depression, a common adverse effect of other analgesic (Phillips et al. 2015). All in all, DEX could be a useful supplement or substitute for conventional painkillers, especially in circumstances where opioids may not be appropriate or acceptable.

The efficacy of this drug as an analgesic and antiemetic is still debated (Oddby‐Muhrbeck et al. 2002), despite its wide use in clinical practice. Compared with placebo, several placebo‐controlled studies show no discernible analgesic effect after DEX administration.

Local DEX wound infiltration was able to shorten hospital stay by requiring a much lower dose of propofol for induction of anesthesia, opioid rescue analgesics requirement, perioperative bleeding, and pain.

Preoperative DEX reduces pain before and after surgery, which in turn reduces the need for inhaled anesthetics or opioid use and avoids PONV.

Thus, preoperative DEX appears to be a promising strategy for managing POP and PONV in patients undergoing bimaxillary orthognathic surgery (BOS), with the added benefit of improving patient satisfaction by limiting the need for rescue analgesics (Labafchi et al. 2023).

Another important consideration in POP management is the recommendation of the ASA to use multimodal analgesia, which combines NSAIDs with local or regional anesthetics. A key aspect of multimodal analgesia is the preoperative administration of NSAIDs. The rationale is to alleviate POP by targeting multiple pain pathways simultaneously. Both opioids and NSAIDs, administered orally or parenterally, are useful in preemptive strategies. In the study by Aoki et al. (2014), all patients received IV catheterization and preoperative chlorhexidine mouth rinse. 30 min before surgery, patients in the ibuprofen group (*n* = 20) received 800 mg IV ibuprofen and 8 mg dexamethasone, while the placebo group (*n* = 20) received sterile IV saline plus 8 mg dexamethasone. Both groups were administered prophylactic IV amoxicillin‐sulbactam (2 g), ondansetron (4 mg), and midazolam (2 mg). The study concluded that a single prophylactic dose of IV ibuprofen significantly reduced VAS pain scores and postoperative opioid use compared with placebo (Canpolat et al. 2021).

The article of Kumar et al. (2023) discusses the risk of PO opioid use disorder PONV and its associated complications in patients undergoing orthognathic surgery. Therefore, it would be very useful to administer an alternative preventive analgesic, such as oral montelukast, in clinical practice to relieve POP.

Montelukast is a specific cysteinyl leukotriene receptor antagonist.

The formation of leukotrienes occurs together with other inflammatory mediators; this increases vascular permeability and promotes migration, aggregation, and degranulation of eosinophils, ultimately contributing to pain.

This study found that the average pain level increased from the first hour after surgery to the 12th hour, when it reached its peak. Thereafter, the pain remained relatively stable and constant until the 18th hour, but decreased thereafter. At all time points, the montelukast group experienced significantly less pain than the placebo group.

This randomized clinical trial involved a total of 60 consecutive individuals.

A single oral dose of 10 mg montelukast was administered preoperatively. Both airflow restriction and nasal symptom scores improved, and the drug markedly reduced inflammatory markers.

Since montelukast reaches its maximum plasma concentration after 3–4 h, administration 1 h before surgery ensures that therapeutic levels are achieved upon emergence from anesthesia.

Before taking the drug, all of the patients had rigid endoscopies. Patients were monitored for an average of 14 weeks, ranging from 6 weeks to 12 months. Montelukast has the potential to greatly reduce headache, nasal discharge, and blockage while also improving scent. Moreover, montelukast lessened the severity of face pain, although the difference was not statistically significant; this can be explained by the follow‐up length and sample size. In conclusion, this study's findings imply that concurrent montelukast treatment can lessen rhinosinusitis symptoms and suffering.

It is possible to draw the conclusion that according to this article preoperative montelukast medication reduces PO discomfort after BOS (Ansari et al. 2022).

In the article of Labafchi et al. (2023) gabapentinoids are discussed. Originally developed as antiepileptic drugs, gabapentinoids act by binding to voltage‐gated calcium channels, thereby reducing hyperalgesia and central sensitization, and are widely used for neuropathic pain management (Qamar et al. 2023). Research has documented the efficaciousness of gabapentin as an analgesic among various surgical populations. Pregabalin is a novel derivative with a better pharmacokinetic profile and efficacy; studies have shown that it can effectively prevent POP at various doses and for various surgical procedures.

Similar to gabapentin in its qualities, pregabalin also possesses dose‐independent absorption and fewer adverse effects (Fassoulaki et al. 2012).

Pregabalin enhances the analgesic impact and reduces the need for opioids when added to the multimodal POP management analgesic strategy. During the surgical phase, non‐steroidal anti‐inflammatory drug dexketoprofen was administered in addition to preemptive pregabalin. Pregabalin was thereby linked to decreased VAS scores as compared to the control group. Additionally, the pregabalin group experienced less rescue analgesia and opioid intake.

This study contains certain limitations. First, the analysis focused exclusively on acute postoperative pain in the context of preventive analgesia; further research is needed to determine whether a single pregabalin dose might also influence the development of chronic pain.

The second limitation is that only one preoperative dose of 150 mg was administered, with no continuation postoperatively. Future studies should therefore investigate pregabalin‐assisted pain prevention both before and after surgery.

Pregabalin 150 mg as a preventive measure reduced the use of PO opioids during the first 24 h of surgery by 48 percent. When compared to a placebo, it produced lower pain scores in the early PO phase. Preemptive analgesia, along with other multimodal analgesia approaches, as also analyzed in the previously analyzed article of Ansari et al. (2022), can be effectively employed in orthognathic surgery to prevent PO discomfort (Ahiskalioglu et al. 2016).

The article of Lee et al. (2017) analyzes tizanidine and its effect in reducing pain post OGS.

Tizanidine is a centrally active α2‐agonist and has demonstrated therapeutic efficacy in the treatment of muscle spasticity. This drug contains analgesic, sedative, and anti‐anxiety qualities. Its structure is similar to that of clonidine. It should be mentioned, however, that people with kidney disease should not take this drug. Accordingly, the study authors did not include patients with kidney disease. After oral administration, tizanidine is readily absorbed in the gastrointestinal tract and reaches maximum plasma concentration 2 h later. Its half‐life is between 2 and 4 h.

Just as is the case when ibuprofen is administered to patients undergoing maxillofacial surgery. Tizanidine administration also reduces the need for opioid medications during and after surgery in the operating room (Canpolat et al. 2021).

1 h before surgery and twice daily for the first week after surgery, patients in the tizanidine group were asked to take one 4‐mg tablet. The control group received the same drug, except an oral placebo was given instead of tizanidine. Tizanidine was found to have the ability to decrease the severity of POP (Shetty and Mohan 2013), reduce the need for analgesics and make it easier for patients to return to their normal lives (Dadmehr 2022).

What was found in the article by Lee and Curtin (2020), was also interesting. Neurosensory assessments and biochemical tests were used. Three patients were randomly assigned and received oral melatonin or placebo for 21 consecutive days as preventive therapy. Subjective pain, numbness and objective neurosensory function were some of the clinical parameters assessed both before and after the intervention, as were serum levels of hydrogen peroxide and antioxidant enzymes.

Melatonin reduced subjective perception of pain by 50% in the first PO days, numbness by 30% after 1 week and by 80% within 3 months after surgery (*p* < 0.00001) (Liu 2016). In the melatonin‐treated group, objective neurosensory tests showed significant improvement in the healing profile. The concentration of hydrogen peroxide (Mehrzadi et al. 2017) in the melatonin group decreased after the intervention, while the levels of antioxidant enzymes increased (*p* < 0.00001). The results of the study indicate that preventive melatonin administration can significantly help reduce POP and improve sensory recovery after surgery (Andersen et al. 2014; Lee and Curtin 2020).

In the article by Kumar et al. (2023), a clinical trial was conducted including 40 subjects (healthy and between the ages of 18 and 45) who were grouped as test and controls. The study was carried out over a full year. The effect of oral montelukast administered before surgery on post‐orthognathic pain levels in class III patients was evaluated. Each patient had to undergo orthognathic BIMAX surgery. The test arm received oral montelukast before the procedure. The pain was measured using the VAS. For every timeline, it was observed that the test and control groups differed significantly from one another. The montelukast group felt significantly less discomfort than the placebo group did at every time point. In terms of pain scores, the placebo group consistently performed better than the montelukast group, even throughout temporal intervals (*p* < 0.05 for all periods). Furthermore, on average, the montelukast group experienced less general discomfort than the placebo group (*p* < 0.001). One may argue that preoperative montelukast treatment contributes to POP reduction in BOS (Kumar et al. 2023).

Instead, in the article of Samieirad et al. (2018), a randomized, triple‐blind clinical trial was conducted. This study recruited healthy systemic patients between the ages of 18 and 45 with skeletal Class III.

60 min before surgery, the first group received an oral dose of 150 μg clonidine tablets dissolved in 20 cc water. The second group also received an 8 mg ondansetron pill dissolved in 20 cc of water at the same time.

Additionally, 60 min before surgery, both groups received an injection of 8 mg of IV dexamethasone. All participants undergo BSSO for mandibular setback and Lefort I osteotomy for maxillary advancement and genioplasty if necessary.

A VAS was used to assess POP. While PO vomiting occurred in the clonidine and ondansetron groups at a rate of 6.7% and 0%, respectively, PO nausea occurred in 53.3% of instances in the ondansetron‐dexamethasone group and in 73.3% of cases in the clonidine‐dexamethasone group. On the other hand, there was no significant difference in the incidence of PO severe pain between the ondansetron and clonidine groups. Nevertheless, the analgesia period was greater with the clonidine group. Therefore, an efficient multimodal approach to minimize postoperative nausea and vomiting and postoperative severe pain in orthognathic operations without serious side effects could be suggested, combining oral ondansetron with IV dexamethasone as premedication (Samieirad et al. 2018; Henzi 2000).

In the study of Xi et al. (2020), it was found that compared with sufentanil, the low incidence of adverse reactions and significant analgesic effects of nalbuphine make it an ideal candidate for pain management after orthognathic surgery. lthough the detailed mechanisms require further investigation (Xi et al. 2020).

Next articles that we will discuss regard cases in which drugs and procedures to limit postoperative pain are administered intraoperatively.

The most commonly used intraoperative and POP therapy involves the use of opioids. Remifentanil, an ultra‐short‐acting μ‐opioid receptor agonist, is often used for intraoperative analgesia because of its rapid onset and effect, allowing for easily controllable analgesia. In addition, at high doses and for a long duration, remifentanil can be used to prevent intraoperative pain without delaying PO recovery or causing respiratory depression. However, exposure to high doses of remifentanil may paradoxically cause hyperalgesia and acute opioid tolerance, which may result in more severe POP and increased need for analgesics (Guignard et al. 2000; Kim et al. 2013; Angst et al. 2003; van Gulik et al. 2012). The mechanism that causes PO hyperalgesia or acute opioid tolerance is still not completely clear Many studies have found that central sensitization through the glutaminergic system and activation of the *N*‐methyl‐d‐aspartate (NMDA) receptor both play an important role in this mechanism (van Gulik et al. 2012; Pasternak 1995; Célèrier et al. 2000; Stubhaug et al. 1997; Rivat et al. 2002). Ketamine is the most common NMDA receptor antagonist (Kanta Kido et al. 2019). They decided to investigate the possible effect of ketamine in controlling remifentanil‐induced (Joly et al. 2005) acute opioid tolerance by analyzing 40 patients undergoing bilateral sagittal reduction of the mandibular ramus. The three groups were given high‐dose remifentanil, low‐dose remifentanil, or high‐dose remifentanil plus ketamine during surgery, respectively. Remifentanil was used as an intraoperative anesthetic in all three groups, and at 24 h PO, the following were analyzed: PO fentanyl use, pain by VAS, NLR, and CRP in serum. The results of this study showed that intraoperative ketamine coadministration increases the NLR in the 24 h after orthognathic surgery and reduces the likelihood of remifentanil‐induced acute PO opioid tolerance without side effects (Beilin et al. 2007). Low‐dose ketamine could therefore help patients undergoing orthognathic surgery avoid acute PO opioid tolerance associated with remifentanil anesthesia (Johnston et al. 2004). The demonstration that the elevated PO NLR induced by ketamine was related to reduced PO opioid consumption could clarify how ketamine prevents remifentanil‐induced acute opioid tolerance. In contrast, CRP levels did not differ significantly among the three study groups. This result is in line with a meta‐analysis that found that cytokine production and ketamine administration did not alter CRP levels (Dale 2012). Finally, thanks to the present study, we can state that in patients receiving remifentanil anesthesia, ketamine may cause an immunosuppressive response instead of an anti‐inflammatory response, affirming the existence of a link between decreased fentanyl consumption associated with increased NLR resulting from ketamine administration. NSAIDs can successfully lower POP and opiate use in patients having BIMAX surgery, according to a study by Seher Orbay Yaşli Jr et al (Orbay Yaşli et al. 2023). When compared to the acetaminophen group, the combination of tenoxicam and paracetamol considerably reduced the level of pain. This shows that patients who woke up from anesthesia with no pain, recovered more quickly, and had lower VAS scores may benefit more from the combination of tenoxicam and acetaminophen for PO analgesia (Ferrara et al. 2022). Aviable alternative to premedications or PO therapies with painkillers is the use of local anesthetics perioperatively and intraoperatively (Quinzi et al. 2020). Spontaneous and evoked nociceptive neuronal activity is inhibited by voltage‐dependent sodium channel blockade of lidocaine, which reduces neuronal hyperactivity, causing PO analgesia. The study by Uilyong Lee Et al. analyzed the trend of pain following BIMAX surgery in 52 patients, divided into two groups, treated with a bolus of 1.5 mg/kg lidocaine followed by a continuous infusion of 2 mg/h/kg during surgery and equal dose of saline, respectively. The VAS to assess pain was used 2, 4, 8, 12, 24 and 40 h after treatment. WBC, neutrophil count, PO edema and total amount of ketorolac consumed were all recorded. In our study, the decrease in pain VAS scores and the usage of rescue analgesics continued for 8 h following surgery. The fact that the analgesic effect lasts longer than the infusion time and plasma half‐life indicates that mechanisms other than sodium channel blockade are at work. One potential additional mechanism that may be involved is the inhibition of glycine uptake by monoethylglycinexylidide, the primary metabolite of lidocaine, which appears to be responsible for preventing central hyperalgesia (Rowland et al. 1971). It has been established that lidocaine exhibits strong anti‐inflammatory properties, reducing the release of cytokines via blocking neutrophil activation (Kawamata et al. 2002). One of the most important phenomena for healing and repair following surgery is inflammation, which results in hemodynamic, endocrine, metabolic, and immunological responses (Herroeder et al. 2002). On the other hand, severe POP and swelling might result from an overreaction to inflammation. Acute phase inflammatory responses are impacted by lidocaine's ability to inhibit lymphocyte proliferation and lower pro‐ and anti‐inflammatory cytokine production (Ferrazzano et al. 2020). This study showed that after BIMAX surgery, intraoperative and preoperative IV lidocaine decreased swelling, the need for rescue analgesics, and POP. Ankita Raj et al. (2021) They conducted a comparative study with the aim of evaluating the differences in efficacy between IM diclofenac administration and local anesthesia in the treatment of pain following maxillofacial surgery (Kim et al. 2016; Perepa et al. 2017). In a trial, 30 patients undergoing BIMAX surgery were split into two groups: group B got injectable diclofenac 75 mg soon before extubation to manage POP, while group A received ropivacaine with bilateral inferior alveolar nerve block. Pain was measured using the VAS scale, and group B patients with a VAS > 5 IM received rescue medication in the form of tramadol HCl 2 mg/kg body weight. Immediately following surgery, a nerve block was given to relieve pain. Those in the nerve block group had VAS scores that were comparable to those of those taking injectable analgesics. For immediate POP management, regional anesthetic is safer than alternative medication administration routes because it avoids the negative side effects of opioids and NSAIDs, which lowers hospital stays and problems. In the PO phase, nerve blocks are administered to ensure anatomical areas are free from discomfort and to prevent needle sticking, particularly in young patients. Regional anesthesia, which consists of nerve blocks administered immediately after surgery, relieves POP, particularly when a long‐lasting local anesthetic agent is used. It also reduces the risk of PO bleeding because the vasoconstrictors in the local anesthetic solution reduce the likelihood of bleeding. Therefore, it is reasonable to state that an inferior alveolar nerve block administered only before extubation is sufficient to reduce the additional amount of analgesic needed for POP management (Lee et al. 2017).

### 5.2 Medications and Procedures for POP Control Administered After the Surgery

The study by Tomic et al. (2022) evaluated the analgesic effect of ibuprofen versus diclofenac plus orphenadrine on POP in orthognathic surgery (Schaffler 2005). Patients were divided into two groups according to the surgery they had undergone. One group received ibuprofen 600 mg and the other group diclofenac 75 mg and orfenadrine 30 mg IVly twice daily. Pain was monitored using a numeric scale General Linear Model (GLM) recorded three times a day from the day of surgery to the next 3 days.

For both groups, repeated GLM measurements showed a significant differenc reduction in NRS pain scores over three consecutive days (*p* < 0.001), but no statistically significant difference between groups (*p* < 0.352).

Group D had a mean NRS of 1.89 (CI 95%, 1.25–2.52) while Group I had a mean NRS of 1.23 (CI 95%, 0.74–1.73) (*p* = 0.104). Nonetheless, in bimaxillary cases, ibuprofen demonstrated superior analgesic efficacy compared with diclofenac plus orphenadrine, providing greater POP relief.

The study by Aoki et al. (2014), records of IV‐controlled analgesia in patients undergoing orthognathic surgery for mandibular prognathism were used to study the time course of fentanyl consumption. In addition, the impact of gender and anesthesia and surgery techniques on time course was examined. Patients were divided into three groups and received fentanyl at three different times of the year.

Fentanyl administration was monitored using a PCA pump, which recorded time, dose, and unsuccessful attempts at self‐administration (“somification”). Following the anesthesia, the dosages were converted into consumption, and a 100 mm VAS was used to calculate the overall intake. A 100 mm scale was used to assess the degree of spontaneous pain in 63 out of 143 instances. After surgery, there were no appreciable variations in total consumption between males and females after 24 h (Nkenke et al. 2012). Based on the anesthetic technique, there were no appreciable differences in consumption between the three groups. On the other hand, compared to the BSSRO group, the bimasculare group's total postoperative consumption was considerably higher. The VAS points and the overall amount of postoperative consumption did not significantly positively correlate. The findings demonstrated that there were differences in total consumption and somification models between the two surgical and three anesthetic techniques. POP also increased as a result of the surgical site's increased number of trigeminal nerve branches.

In Kubota et al.'s (2018) study, an alternative technique was developed for the administration of local anesthetics to control POP in two patients with a respiratory disease. The study used a transdermal drug delivery system, ionophoresis using alternating current with 4% lidocaine (Roustit et al. 2014; Reinauer et al. 1993). An analog scale VAS was used to assess pain severity. A 55‐year‐old woman underwent mandibular wisdom tooth extraction and an 18‐year‐old woman underwent osteotomy of the sagittal ramus of the mandible; both were administered three times at different times, and the VAS always became 0, starting from values of 30 and 22. Thus, this study showed that iontophoresis can reduce POP by reducing the VAS score to zero.

## 6 Limitations

This systematic review has several limitations that should be acknowledged. First, although only randomized controlled trials were included, there was considerable heterogeneity among the studies in terms of surgical procedures, drug classes, dosage regimens, timing of administration, and outcome measures, which prevented a quantitative synthesis and limited the comparability of results. Second, the relatively small sample sizes of several trials may have reduced the statistical power to detect clinically meaningful differences. Third, the methodological quality of some studies was moderate, with risks of bias related to allocation concealment, blinding, or incomplete outcome reporting. Fourth, the follow‐up periods were generally short, focusing mainly on acute postoperative pain, while the potential effect of different analgesic strategies on the transition to chronic pain was rarely assessed. Finally, publication bias cannot be ruled out, as only articles published in English and indexed in the selected databases were considered. These limitations suggest that the present conclusions should be interpreted with caution, and further large‐scale, high‐quality randomized trials with standardized protocols are needed to establish the most effective pharmacological strategies for postoperative pain control in orthognathic surgery.

## 7 Conclusion

The treatment of POP following orthognathic surgery requires a multimodal strategy involving pharmacological agents and procedural interventions. Preoperative administration of medications like DEX, ibuprofen, montelukast, pregabalin, and tizanidine can reduce pain and opioid use. Intraoperative therapies like ketamine combined with remifentanil anesthesia and local anesthetics like lidocaine can prevent early opioid tolerance and reduce pain intensity. Regional anesthetics like nerve blocks can relieve POP without the adverse effects of systemic analgesics. Additional options include NSAIDs like diclofenac and orfenadrine and transdermal drug delivery via iontophoresis. Further studies are needed to investigate the role of medication in managing POP.

## Author Contributions

*Conceptualization*: Gianna Dipalma, Roberta Morolla, and Alessio Danilo Inchingolo. *Methodology*: Pietro Lauria, Roberto Vito Giorgio, and Valeria Colonna. *Software*: Antonio Mancini, Lilla Riccaldo, and Francesco Inchingolo. *Validation*: Antonio ManciniI, Benito F. P. Pennacchio, and Lilla Riccaldo. *Formal analysis*: Roberta Morolla, Antonio Mancini, Roberto Vito Giorgio, and Andrea Palermo. *Investigation*: Gianna Dipalma. *Resources*: Roberto Vito Giorgio and Francesco Inchingolo. *Data curation*: Angelo Michele Inchingolo and Alessio Danilo Inchingolo. *Writing – original draft preparation*: Roberta Morolla, Lilla Riccaldo, and Pietro Lauria. *Writing – review and editing*: Valeria Colonna, Gianna Dipalma, and Angelo Michele Inchingolo. *Visualization*: Alessio Danilo Inchingolo, Antonio Mancini, and Andrea Palermo. *Supervision*: Francesco Inchingolo. *Project administration*: Gianna Dipalma and Francesco Inchingolo. *Funding acquisition*: Francesco Inchingolo and Benito F. P. Pennacchio. All authors have read and agreed to the published version of the manuscript.

## Ethics Statement

The authors have nothing to report.

## Consent

The authors have nothing to report.

## Conflicts of Interest

The authors declare no conflicts of interest.

## Data Availability

All data generated or analyzed during this study are included in this published article.
